# Drought stress affects the protein and dietary fiber content of wholemeal wheat flour in wheat/*Aegilops* addition lines

**DOI:** 10.1371/journal.pone.0211892

**Published:** 2019-02-05

**Authors:** Marianna Rakszegi, Éva Darkó, Alison Lovegrove, István Molnár, László Láng, Zoltán Bedő, Márta Molnár-Láng, Peter Shewry

**Affiliations:** 1 Agricultural Institute, Centre for Agricultural Research, Hungarian Academy of Sciences, Martonvásár, Hungary; 2 Department of Plant Science, Rothamsted Research, Harpenden, United Kingdom; Murdoch University, AUSTRALIA

## Abstract

Wild relatives of wheat, such as *Aegilops* spp. are potential sources of genes conferring tolerance to drought stress. As drought stress affects seed composition, the main goal of the present study was to determine the effects of drought stress on the content and composition of the grain storage protein (gliadin (Gli), glutenin (Glu), unextractable polymeric proteins (UPP%) and dietary fiber (arabinoxylan, β-glucan) components of hexaploid bread wheat (*T*. *aestivum*) lines containing added chromosomes from *Ae*. *biuncialis* or *Ae*. *geniculata*. Both *Aegilops* parents have higher contents of protein and β-glucan and higher proportions of water-soluble arabinoxylans (determined as pentosans) than wheat when grown under both well-watered and drought stress conditions. In general, drought stress resulted in increased contents of protein and total pentosans in the addition lines, while the β-glucan content decreased in many of the addition lines. The differences found between the wheat/*Aegilops* addition lines and wheat parents under well-watered conditions were also manifested under drought stress conditions: Namely, elevated β-glucan content was found in addition lines containing chromosomes 5U^g^, 7U^g^ and 7M^b^, while chromosomes 1U^b^ and 1M^g^ affected the proportion of polymeric proteins (determined as Glu/Gli and UPP%, respectively) under both well-watered and drought stress conditions. Furthermore, the addition of chromosome 6M^g^ decreased the WE-pentosan content under both conditions. The grain composition of the *Aegilops* accessions was more stable under drought stress than that of wheat, and wheat lines with the added *Aegilops* chromosomes 2M^g^ and 5M^g^ also had more stable grain protein and pentosan contents. The negative effects of drought stress on both the physical and compositional properties of wheat were also reduced by the addition of these. These results suggest that the stability of the grain composition could be improved under drought stress conditions by the intraspecific hybridization of wheat with its wild relatives.

## Introduction

Drought is one of the most serious stresses affecting crops, and may reduce the yield production of wheat by up to 50% depending on its frequency and duration [1, 2]. The severity of the effects of drought are particularly acute during the anthesis and grain-filling periods, resulting in decreases in the two major yield components, grain number and grain size [2]. However, drought may also have a considerable effect on the chemical composition of the grain, including the storage protein (gliadins, glutenins) and dietary fiber (arabinoxylan, β-glucan) content and composition [3–5].

Generally, drought stress is known to reduce the carbohydrate content (including sucrose and starch) of the grain [6, 7] and to increase the protein content [8]. However, the effects are highly dependent on the degree and timing of the drought and on interactions with other environmental stresses.

The most important components that determine the breadmaking quality of wheat are the storage proteins: gliadins and glutenins. Prolonged water shortage during the growing season has been found to the increase grain protein content [8]. Recent studies have also shown that the expression of gliadin and glutenin genes may be affected by drought stress as early as three days after anthesis [9]. The amounts of both protein groups are reduced by drought stress, but the magnitude of the effect differs, resulting in decreases in the ratio of glutenins to gliadins and the proportion of unextractable polymeric proteins (UPP%) [7, 10–12]. In agreement with this, drought reduced the glutenin particle size and the proportion of glutenin macropolymers (GMP) in mature grain [13]. In contrast, the concentration of GMP was increased by a single early period of drought, a single late period of drought or combined early drought+late heat stress according to Zhang et al. [14]. These effects could have consequences for processing quality, with Li et al. [15] reporting that drought resulted in increased dough strength but reduced bread volume.

Drought has also been reported to affect the amount and properties of arabinoxylan (AX), the major cell wall polysaccharide and dietary fiber component of wheat and rye. Coles et al. [16] reported that the concentration of AX in wheat was increased by mild drought after flowering but decreased under severe drought, while drought during and after flowering have been reported to increase and decrease the concentration of AX, respectively [17, 18]. By contrast, a recent study reported that drought stress increased the AX content even when applied after flowering [5].

Under natural conditions, drought usually occurs in combination with heat and these two stresses have been reported to act synergistically to increase the dietary fiber content of wheat [19], including AX [5]. The properties and structure of AX may also be affected with the proportion of unsubstituted (US) xylose units being increased under drought stress conditions [5, 20]. Changes in the size and degree of arabinosylation of AX were reported to affect its solubility, and, consequently, its behaviour during processing and impacts on health [21].

β-glucan is the second most abundant cell-wall polysaccharide, and hence dietary fiber component, in wheat (comprising about 25% of the total in white flour), but is the dominant component in barley and oats [22]. A number of studies have reported that drought stress reduces the β-glucan content of barley [23–25] and wheat [5], although Swanston et al. [26] reported the opposite effect, while MacNicol et al. [27] reported that drought applied late in the grain-filling period had no effect on the β-glucan content. In barley the combination of hot and dry conditions resulted in less pronounced effects on the contents of total [26, 28] and water-extractable β-glucan [29] than the single stresses. However, Rakszegi et al [5] reported an opposite trend in wheat, with lower contents of β-glucan and increased proportions of the longer DP5-11 β-glucan units under combined drought+heat stress conditions than under drought or heat, separately.

Improving the stability of grain quality and quantity under drought conditions is an important breeding target [30], with major research programs aimed at developing drought-resistant wheat cultivars [31, 32]. Wild relatives including *Aegilops*, have been proposed as genetic resources for improving the stress tolerance of wheat [33, 34]. Several useful agronomic traits have already been transferred from wild *Aegilops* species to wheat by developing wheat-*Aegilops* hybrids, disomic additions and translocation lines [35, 36]. However, less information is available on the grain quality of wild *Aegilops* species, and on the effects of added chromosomes from these species on the grain composition and quality of wheat [37].

The analysis of several *Aegilops geniculata* accessions showed wide genetic variation at HMW glutenin subunit loci [38, 39]. Medouri et al. [40] identified 27 alleles at the *Glu-M1* and *Glu-U1* loci, resulting in 29 HMW subunit patterns. Similarly, Kozub et al. [41] compared 39 *Aegilops biuncialis* accessions and identified 8 and 10 alleles at the *Glu-U1* and *Glu-Mb1* loci, respectively. Recently, Garg et al. [42] showed that chromosome 1U^g^ negatively affected dough strength in wheat/*Ae*. *geniculata* addition lines, while chromosome 1M^g^ had a positive effect. Rakszegi et al. [43] also compared the grain composition of several *Ae*. *biuncialis* and *Ae*. *geniculata* accessions with bread wheat and showed that they had higher contents of storage proteins and dietary fiber, especially β glucan and soluble AX. Further studies on wheat addition lines involving *Ae*. *biuncialis* and *Ae*. *geniculata* chromosomes showed that *Aegilops* chromosomes from homeologous groups 2, 3, 4, 5 and 7 increased the grain protein under well-watered conditions [43]. Furthermore, the addition of chromosomes 1U^g^ and 1M^g^ increased the proportion of polymeric glutenin proteins, while increased β-glucan content was also observed in addition lines with chromosomes 5U, 7U, and 7M [43]. The addition of chromosomes 5U^g^ and 7M^b^ also affected the structure of wheat AX, as shown by the pattern of oligosaccharides released by digestion with endoxylanase. However, these studies were performed under well-watered growth conditions and no information is available on how the alien chromosomes affect grain composition in a wheat background under drought stress conditions.

Therefore, the main goal of the present work was to study the effects of drought on the content and composition of grain storage protein and dietary fiber components by detailed biochemical analysis of a set of wheat/*Ae*. *geniculata* and wheat/*Ae*. *biuncialis* disomic chromosome addition lines. A further aim was to identify *Aegilops* chromosomes that either improve or stabilise the grain composition of wheat under drought stress.

## Materials and methods

### Plant material and growth conditions

A set of bread wheat (cv. Chinese Spring) / *Ae*. *geniculata* (TA2899) chromosome addition lines and a set of bread wheat (line Mv9kr1) / *Ae*. *biuncialis* (MvGB642) lines were used in experiments together with their wheat and *Aegilops* parental genotypes. The wheat (cv. Chinese Spring) / *Ae*. *geniculata* chromosome addition lines 1U^g^, 2U^g^, 3U^g^, 4U^g^, 5U^g^, 6U^g^, 7U^g^, 1M^g^, 2M^g^, 3M^g^, 5M^g^, 6M^g^ and 7M^g^ were provided by Dr. Bernd Friebe (Kansas State University, Manhattan, Kansas) and maintained by the Cereal Genebank, Martonvásár. The wheat (line Mv9kr1) / *Ae*. *biuncialis* chromosome addition lines 1U^b^, 1U^b^/6U^b^, 3U^b^, 2M^b^, 3M^b^ and 7M^b^ were produced in Martonvásár [44, 45]. The seeds were germinated on wet filter paper in Petri dishes for 3 days at room temperature, and then potted into Jiffy7 pellets (www.jiffygroup.com). The 5-day-old seedlings were vernalised at 4°C for 6 weeks under low light intensity (20 μmolm^-2^s^-1^). After vernalisation, the plants were grown in 2L pots (1 plant/pot) filled with a 3:2:1 mixture of garden soil, compost and sand and placed in completely randomized blocks in a greenhouse (Global Glasshouse Venlo) for 12 weeks, as described by Rakszegi et al. [43]. The growth conditions and water status of the reguralry irrigated plants were similar for all the plants till the flag-leaf sheath extending period (Z41 stage on Zadoks’s scale) [46]. After that, the pots of each genotype were divided into two sets: control plants and drought-treated plants. Each set comprised ten pots per genotype. The control plants were irrigated as before to keep the volumetric soil moisture content (VSMC) between 30 and 35%, where VSMC was measured using an HH2 moisture meter (Delta T device SM-100 sensor, Delta-T Devices Ltd, Cambridge, UK). Drought stress was applied by withholding water and maintaining the average daily VSMC between 10 and 15% for 3 weeks (till the ripening period). This VSMC value represented a non-lethal, but severe drought stress during this period. Drought often occurs in the Central European region before and during wheat flowering and this type of drought treatment is regularly applied to discriminate between drought-sensitive and drought-tolerant genotypes.

### Measurements

The seeds of ten plants per genotype per treatment were harvested separately and seeds from 3 or 4 different plants were bulked to form three biological replicates for analysis. Thousan-kernel weights (TKW) were measured using the standard MSZ 6367/4-86 [47] method. For the determination of grain composition and quality, approximately 4 grams of seed from each biological replicate (3–4 plants) were milled using a Retsch Mixer Mill MM 200 ball mill to produce wholemeal samples, which were immediately cooled and stored at -20°C.

Crude protein content was determined by the Kjeldahl method [48], using a Kjeltec 1035 Analyzer instrument.

Size Exclusion-High Performance Liquid Chromatography (SE-HPLC) was used to determine the glutenin, gliadin and albumin+globulin contents, while the unextractable polymeric protein (UPP% = insoluble glutenin/soluble+insoluble glutenin) content was determined using a modification of the Batey et al. [49] method, as described in Rakszegi et al. [43].

The total amount of mixed-linkage β-glucan was determined according to the protocol of the Megazyme assay kit (Megazyme, Bray, Ireland) [50, 51]. The samples were suspended and hydrated in a buffer solution at pH 6.5 and then incubated with purified lichenase enzyme and filtered. An aliquot of the filtrate was then hydrolysed to completion with purified β-glucosidase. The D-glucose produced was assayed using a glucose oxidase/peroxidase reagent.

Total and water-extractable pentosans, of which AX is the main component, were determined using the colorimetric method reported by Douglas [52] and modified by Finnie et al. [53], as briefly described by Rakszegi et al. [43].

The enzyme fingerprinting of AX and β-glucan was carried out as described by Ordaz-Ortiz et al. [54, 55]. The structure of the AX and β-glucan fiber components was determined after specific enzyme digestion and separation with HP-AEC. The proportions of unsubstituted, mono- and di-substituted xylose residues in the oligosaccharides released from AX (AXOS) and the ratio of DP3 (degree of polymerization) to DP4 units released from β-glucan were calculated from the peak areas [56, 57] and the amounts of monosubstituted (M), disubstituted (D), unsubstituted (US) and total (TOT) AXOS were calculated as decribed in Rakszegi et al. [43].

### Statistical analyses

The experiment was carried out in a greenhouse using a randomized block design in order to minimize the differences caused by the environment. Three biological replicates per each genotype per treatment were used for the analyses. Duplicate analyses were carried out on each biological sample for TKW, protein, β-glucan, pentosan and AX and, if the difference between the two replicate samples was higher than 10%, the measurement was repeated with two more replicates. Three replicate samples were measured when using SE-HPLC or HP-AEC. Statistical analyses were performed as described by Rakszegi et al. [43]. Least Significant Difference (LSD) values between the addition lines, together with the parental wheat and *Aegilops* genotypes, were calculated at the p = 0.05 probability level using the Microsoft Excel program (and used on S1, S2, S3 and S4 Figs). Statistical evaluations were also carried out using SPSS 16.0 software (SPSS Inc., Chicago, IL, USA). The first statistical model applied was the Linear Mixed Model analysis (using the restricted likelihood algorithm, REML) based on Virk et al. [58], where the fixed factors were the genotype (G) and the treatment (T), with replication (R) as the random factor. When the data were split into two groups based on the treatment, G and R were the fixed factors. Tukey’s test was carried out under the Post Hoc multiple pairwise comparisons analysis for observed means, where the fixed factor in the general linear model was the genotype.

## Results

The genotype significantly determined all the compositional quality traits in the studied addition lines and their controls (Table 1). The treatment (drought or control environment) and the interaction of the two factors (GxT) significantly determined the composition of the kernels (protein, β-glucan, arabinoxylan content), but the composition of the different components (Glu/Gli, UPP%, DP3/DP4, US/M+D) was not affected by these factors. There were two properties, UPP% and US/M+D, which were not affected by either the treatment or the GxT interaction.

**Table 1:** Significance of the main effects and their interactions for grain compositional traits tested with Linear Mixed Model analysis.

	Genotype (G)	Treatment (T)	GxT
Thousand-kernel weight (g)	***	***	****
Protein content (mg/g)	***	***	***
Glu/Gli	***	n.s.	***
UPP%	***	n.s.	n.s.
β-glucan content (mg/g)	***	***	***
TOT-pentosan (mg/g)	***	***	***
WE-pentosan (mg/g)	***	*	***
TOT-pentosan/β-glucan	***	***	***
WE/WU-pentosan	***	**	***
TOTAL GOS	***	***	***
DP3/DP4	***	*	n.s.
TOTAL AXOS	***	**	**
US/M+D	*	n.s.	n.s.
M/D	***	***	***

*,**,*** significant at the 0.05, 0.01, 0.001 probability level, respectively

n.s., not significant

AXOS, arabinoxylan oligosaccharides; D, disubstituted AXOS; DP, degree of polymerisation; Gli, gliadin; Glu, glutenin; GOS, glucooligosaccharides; M, monosubstituted AXOS; TOT, total; UPP, unextractable polymeric proteins; US, unsubstituted AXOS; WE, water-extractable; WU, water-unextractable

Separate studies on the effect of genotype and replication under well-watered and drought conditions, showed that the genotype had a more decisive, significant effect on kernel composition than the replication number (Table 2). The replication only affected TKW, protein and β-glucan content, while the genotype determined all the properties under both well-watered and drought conditions (with one exception under drought).

**Table 2:** Significance of main effects and their interactions for grain compositional traits split by treatment and tested using Linear Mixed Model analysis.

	Genotype	Replication	Genotype	Replication
	control	control	drought	drought
Thousand-kernel weight (g)	***	***	***	***
Protein content (mg/g)	***	***	***	***
Glu/Gli	***	n.s.	***	n.s.
UPP%	***	n.s.	***	n.s.
β-glucan content (mg/g)	***	*	***	n.s.
TOT-pentosan (mg/g)	***	n.s.	***	n.s.
WE-pentosan (mg/g)	***	n.s.	***	n.s.
TOT-pentosan/β-glucan	***	n.s.	***	*
WE/WU-pentosan	***	n.s.	***	n.s.
TOTAL GOS	***	n.s.	***	***
DP3/DP4	***	***	*	n.s.
TOTAL AXOS	***	n.s.	***	n.s.
US/M+D	***	n.s.	n.s.	n.s.
M/D	***	n.s.	***	n.s.

*,**,*** significant at the 0.05, 0.01, 0.001 probability level, respectively

n.s., not significant

AXOS, arabinoxylan oligosaccharides; D, disubstituted AXOS; DP, degree of polymerisation; Gli, gliadin; Glu, glutenin; GOS, glucooligosaccharides; M, monosubstituted AXOS; TOT, total; UPP, unextractable polymeric proteins; US, unsubstituted AXOS; WE, water-extractable; WU, water-unextractable

In addition to the overall effects, however, it is also important to investigate the effect of drought on the compositional properties of individual lines and to carry out pairwise comparisons.

### Effect of drought on the protein content and composition of wheat/*Aegilops* addition lines

The thousand-kernel weights (TKW) of *Ae*. *geniculata* and *Ae*. *biuncialis* were significantly lower than those of the wheat parents (Chinese Spring and Mv9kr1) under drought conditions, while some of the addition lines (1U^g^, 7U^g^, 1M^g^, 6M^g^, 7M^g^ and 7M^b^) had significantly higher TKW than the relevant drought-treated wheat genotype (Table 3, S1 Fig). Drought stress itself resulted in a significant decrease in the TKW of wheat/*Aegilops* addition lines containing group 3 or 4 chromosomes from the U^g^ genome of *Ae*.*geniculata* and in an increase in the 1M^g^ addition line, as compared to their untreated, well-watered controls (Table 4).

**Table 3:** Comparison of the effects of different chromosomes in a wheat (*T*. *aestivum*) genetic background under drought conditions using the Post Hoc multiple pairwise comparison test. Orange and blue blocks indicate values higher and lower than the control, respectively.

(I) Genotype	(J) Genotype	TKW	Protein	Glu/Gli	UPP%	β-glucan	TOT- pentosan	WE- pentosan	TOT-pentosan/ β-glucan	WE/WU- pentosan	TOTAL GOS	DP3/DP4	TOTAL AXOS	US/M+D	M/D
dCS	dAe.gen	*	*	***	***	***	***	n.s.	***	**	***	n.s.	***	*	***
	dAeg 1U	*	n.s.	n.s.	n.s.	n.s.	n.s.	***	***	n.s.	n.s.	n.s.	n.s.	n.s.	n.s.
	dAeg 2U	n.s.	n.s.	**	n.s.	n.s.	n.s.	***	***	n.s.	**	n.s.	n.s.	n.s.	n.s.
	dAeg 3U	n.s.	n.s.	n.s.	n.s.	n.s.	***	***	***	n.s.	n.s.	n.s.	n.s.	n.s.	*
	dAeg 4U	n.s.	n.s.	n.s.	n.s.	n.s.	n.s.	***	***	n.s.	n.s.	n.s.	n.s.	n.s.	n.s.
	dAeg 5U	n.s.	n.s.	n.s.	n.s.	*	**	**	***	n.s.	n.s.	n.s.	n.s.	n.s.	n.s.
	dAeg 6U	n.s.	n.s.	n.s.	n.s.	n.s.	***	***	***	n.s.	**	n.s.	n.s.	n.s.	n.s.
	dAeg 7U	*	n.s.	n.s.	n.s.	***	***	***	***	n.s.	***	n.s.	n.s.	n.s.	n.s.
	dAeg 1M	***	n.s.	***	***	n.s.	***	***	***	n.s.	n.s.	n.s.	n.s.	n.s.	n.s.
	dAeg 2M	n.s.	n.s.	n.s.	n.s.	n.s.	***	***	***	n.s.	*	n.s.	n.s.	n.s.	n.s.
	dAeg 3M	n.s.	n.s.	n.s.	n.s.	n.s.	***	***	***	n.s.	n.s.	n.s.	n.s.	n.s.	n.s.
	dAeg 5M	n.s.	n.s.	*	n.s.	n.s.	***	***	***	n.s.	n.s.	n.s.	n.s.	n.s.	n.s.
	dAeg 6M	**	n.s.	n.s.	n.s.	n.s.	***	***	***	n.s.	n.s.	n.s.	n.s.	n.s.	n.s.
	dAeg 7M	*	n.s.	n.s.	n.s.	***	***	***	***	n.s.	***	n.s.	n.s.	n.s.	n.s.
dMv9kr1	dAe.biu	***	***	***	***	***	***	n.s.	***	***	***	n.s.	*	*	*
	dAebiu1U	n.s.	n.s.	***	n.s.	n.s.	n.s.	***	n.s.	n.s.	n.s.	n.s.	n.s.	n.s.	n.s.
	dAebiu16U	n.s.	n.s.	***	n.s.	n.s.	***	***	***	n.s.	*	n.s.	n.s.	n.s.	n.s.
	dAebiu3U	n.s.	n.s.	n.s.	n.s.	n.s.	*	n.s.	n.s.	n.s.	n.s.	n.s.	n.s.	n.s.	n.s.
	dAebiu2M	n.s.	n.s.	n.s.	n.s.	n.s.	n.s.	n.s.	n.s.	n.s.	n.s.	n.s.	n.s.	n.s.	***
	dAebiu3M	n.s.	n.s.	n.s.	n.s.	n.s.	n.s.	*	n.s.	n.s.	n.s.	n.s.	n.s.	*	***
	dAebiu7M	**	n.s.	n.s.	n.s.	***	n.s.	n.s.	***	n.s.	***	n.s.	n.s.	n.s.	**

*,**,*** significant at the 0.05, 0.01, 0.001 probability level, respectively

n.s., not significant; d, drought-treated samples

AXOS, arabinoxylan oligosaccharides; D, disubstituted AXOS; DP, degree of polymerisation; Gli, gliadin; Glu. glutenin; GOS, glucooligosaccharides; M, monosubstituted AXOS; TOT, total; UPP, unextractable polymeric proteins; US, unsubstituted AXOS; WE, water-extractable; WU, water-unextractable

**Table 4:** Comparison of drought-induced changes in grain composition for each genotype using the Post Hoc multiple pairwise comparison test. Orange and blue blocks indicate values higher and lower than the control, respectively.

(I) Genotype	(J) Genotype	TKW	Protein	Glu/Gli	UPP%	β-glucan	TOT- pentosan	WE- pentosan	TOT-pentosan/ β-glucan	WE/WU- pentosan	TOTAL GOS	DP3/DP4	TOTAL AXOS	US/M+D	M/D
CS	dCS	n.s.	***	n.s.	n.s.	***	***	***	***	n.s.	*	n.s.	n.s.	n.s.	n.s.
Ae.gen	dAe.gen	n.s.	n.s.	***	n.s.	***	n.s.	***	n.s.	n.s.	n.s.	n.s.	n.s.	n.s.	n.s.
Aeg 1U	dAeg 1U	n.s.	***	***	n.s.	n.s.	***	***	**	n.s.	n.s.	n.s.	n.s.	n.s.	n.s.
Aeg 2U	dAeg 2U	n.s.	n.s.	***	n.s.	n.s.	***	***	***	n.s.	n.s.	n.s.	n.s.	n.s.	n.s.
Aeg 3U	dAeg 3U	**	**	n.s.	n.s.	n.s.	n.s.	n.s.	*	n.s.	n.s.	n.s.	n.s.	n.s.	n.s.
Aeg 4U	dAeg 4U	**	n.s.	n.s.	n.s.	n.s.	***	n.s.	*	n.s.	n.s.	n.s.	n.s.	n.s.	n.s.
Aeg 5U	dAeg 5U	n.s.	n.s.	n.s.	n.s.	***	*	**	n.s.	n.s.	n.s.	n.s.	n.s.	n.s.	n.s.
Aeg 6U	dAeg 6U	n.s.	***	**	n.s.	n.s.	n.s.	n.s.	n.s.	n.s.	n.s.	n.s.	n.s.	n.s.	n.s.
Aeg 7U	dAeg 7U	n.s.	*	n.s.	n.s.	***	n.s.	*	n.s.	n.s.	n.s.	n.s.	n.s.	n.s.	n.s.
Aeg 1M	dAeg 1M	***	***	n.s.	n.s.	n.s.	n.s.	n.s.	n.s.	n.s.	n.s.	n.s.	n.s.	n.s.	n.s.
Aeg 2M	dAeg 2M	n.s.	n.s.	n.s.	n.s.	n.s.	n.s.	n.s.	n.s.	n.s.	n.s.	n.s.	n.s.	n.s.	n.s.
Aeg 3M	dAeg 3M	n.s.	***	*	n.s.	***	n.s.	*	*	n.s.	*	n.s.	n.s.	n.s.	n.s.
Aeg 5M	dAeg 5M	n.s.	n.s.	n.s.	n.s.	n.s.	n.s.	n.s.	n.s.	n.s.	n.s.	n.s.	n.s.	n.s.	n.s.
Aeg 6M	dAeg 6M	n.s.	***	***	n.s.	n.s.	n.s.	**	n.s.	n.s.	n.s.	n.s.	n.s.	n.s.	n.s.
Aeg 7M	dAeg 7M	n.s.	n.s.	n.s.	n.s.	n.s.	n.s.	***	n.s.	n.s.	n.s.	n.s.	n.s.	n.s.	n.s.
Mv9kr1	dMv9kr1	n.s.	***	n.s.	n.s.	*	**	*	***	n.s.	***	n.s.	n.s.	n.s.	n.s.
Ae.biu	dAe.biu	n.s.	n.s.	n.s.	n.s.	n.s.	n.s.	n.s.	n.s.	n.s.	n.s.	n.s.	n.s.	n.s.	n.s.
Aebiu 1U	dAebiu1U	n.s.	***	n.s.	n.s.	n.s.	n.s.	n.s.	n.s.	n.s.	n.s.	n.s.	n.s.	n.s.	n.s.
Aebiu16U	dAebiu16U	n.s.	***	**	n.s.	n.s.	n.s.	*	n.s.	n.s.	n.s.	n.s.	n.s.	n.s.	n.s.
Aebiu 3U	dAebiu3U	n.s.	***	n.s.	n.s.	n.s.	n.s.	n.s.	n.s.	n.s.	n.s.	n.s.	n.s.	n.s.	n.s.
Aebiu 2M	dAebiu2M	n.s.	***	n.s.	n.s.	n.s.	n.s.	**	n.s.	n.s.	n.s.	n.s.	n.s.	n.s.	***
Aebiu 3M	dAebiu3M	n.s.	***	n.s.	n.s.	*	n.s.	***	**	***	**	n.s.	***	***	n.s.
Aebiu 7M	dAebiu7M	n.s.	**	n.s.	n.s.	n.s.	***	***	***	n.s.	n.s.	n.s.	n.s.	n.s.	n.s.

*,**,*** significant at the 0.05, 0.01, 0.001 probability level, respectively; d, drought-treated samples

AXOS, arabinoxylan oligosaccharides; D, disubstituted AXOS; DP, degree of polymerisation; Gli, gliadin; Glu, glutenin; GOS, glucooligosaccharides; M, monosubstituted AXOS; TOT, total; UPP, unextractable polymeric proteins; US, unsubstituted AXOS; WE, water-extractable; WU, water-unextractable.

The protein content was higher in the *Aegilops* accessions than in the wheat parents, but there were no significant differences between the addition lines and the wheat parents in the case of drought (Table 3, S1 Fig). Although drought stress resulted a significant increase in the protein contents of the parental wheat genotypes (Table 5), the values were still below those of the *Aegilops* accessions (S1 Fig). The protein contents of addition lines 1U^g^, 3U^g^, 6U^g^, 7U^g^, 1M^g^, 3M^g^, 6M^g^, 1U^b^, 1U/6U^b^, 2M^b^, 3M^b^ and 7M^b^ significantly increased as a result of drought stress (Table 5).

**Table 5:** Comparison of the effects of different chromosomes in a wheat (*T*. *aestivum*) genetic background under well-watered conditions using the Post Hoc multiple pairwise comparison test. Orange and blue blocks indicate values higher and lower than the control, respectively.

(I) Genotype	(J) Genotype	TKW	Protein	Glu/Gli	UPP%	β-glucan	TOT- pentosan	WE- pentosan	TOT-pentosan / β-glucan	WE/WU- pentosan	TOTAL GOS	DP3/DP4	TOTAL AXOS	US/M+D	M/D
CS	Ae.gen	***	***	***	***	***	n.s.	n.s.	***	*	***	n.s.	***	***	***
	Aeg 1U	n.s.	n.s.	**	n.s.	n.s.	n.s.	n.s.	n.s.	n.s.	n.s.	n.s.	n.s.	n.s.	n.s.
	Aeg 2U	n.s.	***	n.s.	n.s.	n.s.	n.s.	***	n.s.	**	n.s.	n.s.	n.s.	n.s.	n.s.
	Aeg 3U	n.s.	n.s.	n.s.	n.s.	n.s.	n.s.	n.s.	n.s.	n.s.	n.s.	n.s.	n.s.	n.s.	n.s.
	Aeg 4U	n.s.	***	n.s.	n.s.	n.s.	n.s.	n.s.	n.s.	n.s.	n.s.	n.s.	n.s.	n.s.	*
	Aeg 5U	n.s.	***	**	n.s.	***	***	***	n.s.	n.s.	n.s.	n.s.	n.s.	n.s.	*
	Aeg 6U	n.s.	n.s.	n.s.	n.s.	n.s.	n.s.	n.s.	n.s.	n.s.	n.s.	n.s.	n.s.	n.s.	n.s.
	Aeg 7U	n.s.	**	n.s.	n.s.	**	*	n.s.	n.s.	n.s.	n.s.	n.s.	n.s.	n.s.	n.s.
	Aeg 1M	n.s.	n.s.	n.s.	***	n.s.	n.s.	n.s.	n.s.	n.s.	n.s.	n.s.	n.s.	n.s.	n.s.
	Aeg 2M	n.s.	***	***	n.s.	n.s.	n.s.	n.s.	n.s.	n.s.	n.s.	n.s.	n.s.	n.s.	n.s.
	Aeg 3M	n.s.	n.s.	n.s.	n.s.	n.s.	n.s.	n.s.	n.s.	n.s.	n.s.	n.s.	n.s.	n.s.	n.s.
	Aeg 5M	n.s.	***	n.s.	n.s.	n.s.	n.s.	***	n.s.	n.s.	***	n.s.	n.s.	n.s.	n.s.
	Aeg 6M	n.s.	n.s.	n.s.	n.s.	n.s.	n.s.	*	n.s.	n.s.	n.s.	n.s.	n.s.	n.s.	n.s.
	Aeg 7M	n.s.	***	*	n.s.	n.s.	n.s.	*	n.s.	n.s.	*	n.s.	n.s.	n.s.	n.s.
Mv9kr1	Ae.biu	***	***	***	***	***	n.s.	n.s.	***	*	***	n.s.	*	***	n.s.
	Aebiu 1U	n.s.	n.s.	***	n.s.	n.s.	n.s.	n.s.	n.s.	n.s.	**	n.s.	n.s.	n.s.	n.s.
	Aebiu16U	n.s.	n.s.	***	n.s.	n.s.	n.s.	n.s.	n.s.	n.s.	n.s.	n.s.	n.s.	n.s.	n.s.
	Aebiu 3U	n.s.	*	n.s.	n.s.	n.s.	n.s.	n.s.	n.s.	n.s.	n.s.	n.s.	n.s.	n.s.	n.s.
	Aebiu 2M	n.s.	***	n.s.	n.s.	n.s.	n.s.	***	***	***	*	n.s.	n.s.	n.s.	***
	Aebiu 3M	n.s.	*	n.s.	n.s.	n.s.	n.s.	***	n.s.	***	***	n.s.	n.s.	n.s.	***
	Aebiu 7M	n.s.	***	n.s.	**	**	n.s.	***	***	n.s.	n.s.	n.s.	**	*	**

*,**,*** significant at the 0.05, 0.01, 0.001 probability level, respectively

n.s., not significant

AXOS, arabinoxylan oligosaccharides; D, disubstituted AXOS; DP, degree of polymerisation; Gli, gliadin; Glu, glutenin; GOS, glucooligosaccharides; M, monosubstituted AXOS; TOT, total; UPP, unextractable polymeric proteins; US, unsubstituted AXOS; WE, water-extractable; WU, water-unextractable

The ratio of polymeric glutenin to monomeric gliadin storage proteins **(**Glu/Gli), which determines dough strength and extensibility, was significantly higher in *Ae*. *geniculata*, but lower in *Ae*. *biuncialis* than in the wheat parents during drought (Table 3, S1 Fig). Higher Glu/Gli ratios were also found in wheat/*Ae*. *geniculata* addition lines containing the 2U^g^, 1M^g^ and 5M^g^ chromosomes, but the ratios were significantly lower ratios in wheat/*Ae*. *biuncialis* addition lines with chromosomes 1U^b^ and 1U/6U^b^ (Table 3). Compared to the unstressed controls, the Glu/Gli ratio was not affected by drought stress in most of the addition lines, but there was an increase in the *Ae*. *geniculata* accession and the 2U^g^ addition line and a decrease in lines 1U^g^, 6U^g^, 3M^g^, 6M^g^ and 1/6U^b^ (Table 4).

The ratio of unextractable polymeric proteins (UPP%), which is related to dough strength, varied in a similar fashion to the Glu/Gli ratio, but greater differences were observed between the genotypes (Table 3). When exposed to drought the *Ae*. *geniculata* accession and the 1M^g^ addition line had significantly higher UPP% than the wheat parental control (Table 3), but no significant differences were observed in the UPP% of drought-stressed addition lines or their parents compared to their untreated controls (Table 4).

### Effect of drought on the content and composition of dietary fiber in the wheat/*Aegilops* addition lines

The greatest difference in dietary fiber components between the wheat and *Aegilops* genotypes was in the β-glucan content, which was 4 to 8 times higher under drought stress (Table 3, S2 Fig). Higher β-glucan content was also observed in addition lines with the 5U^g^, 7U^g^, and 7M chromosomes compared to the parental wheat genotypes under drought stress conditions (Table 3). Drought stress resulted in decreased β-glucan content in many genotypes, particularly in the wheat parents, *Ae*. *geniculata* and in addition lines with chromosomes 5 U^g^, 7U^g^, 3M^g^ and 3M^b^ (Table 4).

The highest contents of total pentosans (TOT-pentosan), of which the major component is AX, were found in the wheat parents, with the *Aegilops* parents and most of the addition lines having significantly lower TOT-pentosan contents during drought compared to wheat (Table 3, S2 Fig). Compared to the unstressed control plants, the TOT-pentosan content increased significantly in wheat, but did not change in the *Aegilops* parents. The TOT-pentosan contents of addition lines with chromosomes 1U^g^, 2U^g^, 4U^g^ and 7M^b^ also increased under drought stress, but decreased in line 5U^g^ (Table 4).

The water-extractable pentosan (WE-pentosan) content was similar in the *Aegilops* and wheat parents under drought conditions (Table 3, S2 Fig), while almost all of the addition lines had significantly lower WE-pentosan content compared to wheat when subjected to drought (Table 3). Drought stress resulted in a greater increase in WE-pentosans in the wheat lines than in the *Aegilops* accessions (Table 4). Furthermore, increased contents of WE-pentosan were observed in addition lines with chromosomes 1U^g^, 2U^g^, 3M^g^ and 6M^g^, and decreases in lines with chromosomes 5U^g^, 7U^g^, 7M^g^, 1U/6U^b^, 2M^b^, 3M^b^ and 7M^b^ due to drought stress (Table 4).

The ratio of TOT-pentosans to β-glucan was ten to twelve times higher in wheat than in the *Ae*. *geniculata* or *Ae*. *biuncialis* accessions in response to drought (S3 Fig). All the *Ae*. *geniculata* addition lines and *Ae*. *biuncialis* addition lines with chromosomes 1U/6U^b^ and 7M^b^ had lower ratios of TOT-pentosans to β-glucan than wheat in the case of drought (Table 3), but they still had about 4-fold higher ratios than *Aegilops*. Compared to the well-watered control plants (Table 4) the ratio of TOT-pentosans to β-glucan was higher in many drought-stressed plants. The greatest increase was observed in the wheat parents and in the chromosome 1-4U^g^, 3M^g^, 3M^b^ and 7M^b^ addition lines (Table 4).

The ratio of water-extractable to unextractable pentosans (WE/WU-pentosan) was significantly higher in the *Ae*. *geniculata* and *Ae*. *biuncialis* accessions than in wheat under drought stress (Table 3, S3 Fig). The WE/WU ratio in all the addition lines was similar to that of wheat under drought conditions (Table 3). Compared with the well-watered control plants, the WE/WU ratio was lower in the 3M^b^ addition line as a result of drought (Table 4).

Changes in the structure of the AX and β-glucan fiber components were determined by comparing the oligosaccharides released by specific enzyme digestion, separated using HP-AEC. The major glucooligosaccharides (GOS) released from β-glucan by digestion with lichenase comprise 3 (DP3) or 4 (DP4) glucose units, reflecting the number and distribution of (1→3) and (1→4) linkages within the polymers. The total GOS value is therefore related to the total content of β-glucan, while the ratio of DP3/DP4 GOS provides information on the structure.

As in the case of the β-glucan content (measured using a commercial kit), the TOTAL GOS content of the *Aegilops* lines was significantly higher than that of the wheat lines under drought stress (Table 3, S4 Fig). Many addition lines also had higher TOTAL GOS contents compared than the wheat parents during drought including those with chromosomes 2U^g^, 6U^g^, 7U^g^, 2M^g^, 7M^g^ from *Ae*. *geniculata*, and 1/6U^b^ and 7M^b^ from *Ae*. *biuncialis* under drought (Table 3, S4 Fig). Drought stress resulted in a significant decrease in the TOTAL GOS content in the wheat parents and in addition lines 3M^g^ and 3M^b^ compared to their unstressed control plants (Table 4).

The DP3/DP4 ratio was similar in the different genotypes under drought conditions compared to the wheat control (Table 3, S4 Fig) and was not affected by drought stress compared to the unstressed control plants (Table 4).

The digestion of AX using a specific endoxylanase results in the release of a range of AXOS, the total amount of which (TOTAL AXOS) reflects the content of total AX. The *Aegilops* species had lower TOTAL AXOS than wheat during drought stress (Table 4), as also found for the TOT-pentosan content, but no differences were observed in the TOTAL AXOS contents of the addition lines (Table 3, S4 Fig). Compared to the untreated controls, drought resulted in a decrease in the 3M^b^ addition line (Table 4).

Previous studies have characterized the AXOS released by endoxylanase digestion [59], showing the presence of unsubstituted arabinosylated xylose units and of units mono- or di-substituted with arabinose. Using the relative values from the enzymatic digestions, it is possible to calculate the approximate ratio of unsubstituted AXOS to mono- and di-substituted AXOS units (US/M+D) and the ratio of the mono- to di-substituted AXOS units (M/D). The data presented in S4 Fig (Table 3) showed that the level of substitution was lower in *Aegilops* than in wheat under drought. In addition, the US/M+D ratio was higher in the 3M^b^ addition lines under drought conditions (Table 3, S4 Fig). Drought had a significant effect on the US/M+D ratio of the 3M^b^ addition line (Table 4). The M/D ratio was higher in *Aegilops* and in addition lines with chromosomes 2M^b^, 3M^b^ and 7M^b^ under drought stress, while the M/D ratio was lower in line 3U^g^ (Table 3, S4 Fig). Drought did not affect the M/D ratio, except in line 2M^b^ (Table 4).

## Discussion

The effect of drought on grain composition and quality was studied in wheat/*Aegilops* addition lines in order to evaluate their usefulness as genetic sources of high quality under drought stress for exploitation in bread wheat. The results are compared with the results found under well-watered conditions [43] (Table 5).

### Drought-induced changes in grain composition and quality

It is well known that drought stress causes considerable changes in grain composition, including a substantial decrease in starch accumulation [6, 60] and an increase in grain protein content [8, 11]. However, drought can also affect both the protein composition [7, 61] and the structure of the major dietary fiber components (β-glucan and AX) [5].

The present study showed that the amounts of total proteins and TOT-pentosans (mainly AX) increased in wheat under drought stress, while the β-glucan content decreased, in agreement with previous findings [5]. These changes influenced TOT-pentosan/β-glucan ratio, which increased in response to drought stress, while the arabinosylation level of AX (US/M+D) slightly (but not significantly) decreased. The lower level of arabinosylation can be expected to reduce the solubility of the molecules, if there is an increase or no change in the number of crosslinks.

In the *Aegilops* species, however, these effects were less marked or absent. This may relate to the relative sizes of wheat and *Aegilops* grains, the latter being much smaller with a lower starch content. Hence, as drought primarily influences starch accumulation, the knock-on effects of decreased starch accumulation on the proportions of proteins and fiber components would be less in *Aegilops*. The *Aegilops* species also differ from wheat in their dietary fiber composition, having high proportions of β-glucans and low proportions of AX (as also observed in barley and oats) [62, 63].

Previously reported studies on the effect of drought on wheat composition are partly consistent with these results on *Aegilops*, showing decreases in the ratio of glutenin to gliadin components and in the proportion of unextractable polymeric proteins (UPP%) [7, 10– 12]. Dai et al. [13] reported that drought reduced glutenin particle size and the proportion of glutenin macropolymers (GMP) in mature grain. However, the present analysis showed that the UPP% of the addition lines was not affected by drought, while the Glu/Gli ratio decreased in the 1U^g^, 6U^g^, 3M^g^, 6M^g^ and 1/6U^b^ addition lines, indicating an increased proportion of monomeric gliadins. However, the addition line containing chromosome 2U^g^ had higher levels of UPP%. These changes would be expected to affect the strength of the gluten and dough.

It is known that the AX concentration depends on the severity of the drought stress, increasing after mild drought but decreasing under severe stress [16]. In our study, the content of TOT-pentosan (mostly AX) and the ratio of unsubstituted AXOS (US/M+D) increased in several addition lines under drought stress conditions, but, in the latter case, this increase was only significant in the 3M^b^ addition line. The effects of the environment on the β-glucan content of cereals have mainly been studied in barley [25], where it is the major cell-wall polysaccharide. Coles et al. [23] found a relationship between β-glucan synthesis and transpiration, with low β-glucan content occurring in environments such as drought stress. This is supported by the results of Narasimhalu et al. [24] and Rakszegi et al. [5], who also found that drought decreased the β-glucan content in wheat, as found here in many addition lines. By contrast, Swanston et al. [26] reported higher β-glucan contents in barley grown in Spain (which was hot and dry) than in the cool and wet climate of Scotland, suggesting that the total β-glucan and WE β-glucan contents depend on the timing and degree of stress and are greater under hot, dry conditions, while a short, very severe period of high temperature stress may reduce the β-glucan content [26–29, 64].

#### Effect of *Aegilops* chromosomes on grain quality and composition under drought stress conditions

It was previously shown that the addition of various U- and M- genome chromosomes from *Aegilops* may affect the protein and fiber content and composition of wheat grain [43]. However, no information has previously been published on the effect of these chromosomes on wheat grain composition and quality under abiotic stress conditions. Although Zhou et al. [65] identified proteins involved in the protection against drought stress in wheat-*Ae*. *longissima* 1S(1B) substitution lines, these proteins were not related to grain quality.

Changes in grain yield, kernel number per spike or thousand-kernel weight were widely studied in wheat introgression lines and significant improvement was achieved using introgressed rye (1RS.1BL) [66] or *Agropyron cristatum* (6P) chromosome segments [67, 68].

Rakszegi et al. [43] reported higher thousand-kernel weight (TKW) in a number of wheat-*Aegilops* addition lines (based on LSD values), including those with chromosomes 1U^g^, 1M^g^ and 7M^g^, under well-watered control conditions (Table 5). It has now been shown that these increases also occurred under drought stress conditions (Table 3, S1 Fig). On the other hand, chromosomes 7U^g^, 6M^g^ and 7M^b^ helped to stabilize kernel size under adverse drought stress conditions, so drought caused no change in this parameter in these lines (Table 3, S1 Fig).

The protein content was also widely studied in introgression lines of wheat and was found to be improved by rye chromosomes 4R and 6R [69, 70], the E or F chromosomes of *Aegilops caudata* [71], substitution and translocation lines involving *Thinopyrum intermedium* ssp. *trichophorum* chromosome 1St#2 [72], the ADL.1U(b) disomic addition line of *Aegilops biuncialis* [73] or substitution lines containing *Triticum turgidum* subsp. *dicoccoides* chromosome arms [74]. The grain protein content of many *Ae*. *geniculata* and *Ae*. *biuncialis* accessions, including the *Aegilops* parents used in this experiments was higher than that of many wheat genotypes when the plants were grown under well-watered conditions [43]. In the present study the analysis of wholemeal samples showed much higher contents of protein (2.5 mg/g) and β-glucan (30–50 mg/g) and higher proportions of WE-pentosans (0.3 WE/WU) in *Aegilops* than in wheat under drought stress condition (S1 Fig, S2 Fig). The higher contents of protein and β-glucan presumably reflect the smaller grain size and lower starch content, as these would result in lower dilution of the protein with starch, and a higher proportion of fiber-rich outer bran layers.

The protein content was also higher in addition lines involving chromosomes 2U^g^, 4U^g^, 5U^g^, 7U^g^, 2M^g^ 5M^g^ 7M^g^, 3U^b^, 2M^b^ 3M^b^ and 7M^b^ under well-watered conditions [43], but these increases were not observed under drought stress (Table 3, S1 Fig, Table 5). This may be due to the fact that the drought-induced increase in protein content was more intense in plants with lower protein content under well-watered conditions than in those with elevated protein content. However, the protein composition of the addition lines differed both under control and drought stress conditions, as indicated by the different values of Gli/Glu ratio and UPP%.

Increased Glu/Gli ratios were observed in addition lines with chromosomes 2U^g^, 1M^g^ and 5M^g^ under drought stress conditions, but only the 1U^g^ line showed a similar effect under well-watered conditions (Table 3, S1 Fig, Table 5). The unextractable polymeric protein (UPP%) content was less affected by the chromosome additions or by drought. but was higher in the 1M^g^ lines under both well-watered and drought conditions. A higher Glu/Gli ratio under drought stress may result in greater gluten and dough strength, together with increased water absorption by the flour. However, the Glu/Gli ratio was lower in *Ae*. *biuncialis* and in addition lines with chromosomes 1U^b^ and 1U/6U^b^ than in wheat, under both drought and well-watered conditions.

Studies on the dietary fibers in wheat introgression lines revealed that group-7 long-arm wheat-rye Robertsonian translocations resulted in higher β-glucan content in wheat [75]. The greatest difference between the wheat and *Aegilops* parents was in their β-glucan contents, which was significantly higher in *Aegilops* than in wheat particularly under drought stress, which resulted in 4 to 8 times higher β-glucan contents in *Aegilops* than in wheat. A significantly higher β-glucan content was also observed in several addition lines (5U^g^, 7U^g^ and 7M^b^), than in the wheat parents under both well-watered and drought stress conditions (Table 3, S2 Fig, Table 5). Similar effects were observed for 7M^g^ under well-watered conditions and for 7U^g^, 7M^g^ and 7M^b^ under drought stress when β-glucan was determined as TOTAL GOS using HP-AEC (S4 Fig), but the Megazyme method is likely to be more accurate. Chromosomes 2U^g^, 6U^g^, 2M^g^ and 1/6U^b^ also had a significant effect on TOTAL GOS in the case of drought. Several genes that affect the accumulation of β-glucan have been identified on chromosomes 1U, 2U, 5U, 6U and 7U of *Aegilops umbellulata* (as discussed by Rakszegi et al. [43]), so the same chromosomes in *Ae*. *geniculata* may contribute to the increased β-glucan content in the addition lines under drought stress conditions.

The structure of β-glucan, determined by the ratio of DP3 to DP4 GOS released by enzymatic digestion, only differed in line 5U^g^ under both drought and well-watered conditions, with a ratio that was slightly lower than in wheat (S4 Fig). A higher ratio of longer (DP4) β-glucan units would be expected to result in lower solubility of the molecules. Unsoluble fibers help to improve the condition of the bowels but may have negative effects on processing. However, the effects of these quantitative changes in the fiber properties may not be detectable in practice at all.

One of the most significant fibers component of wheat is arabinoxylan. Recent studies showed that the introgression of rye chromosomes 4R [69], 1R and 6R [70] into wheat significantly increased the total arabinoxylan content [69]. While both the TOT- and WE-pentosan contents increased in several wheat-*Aegilops* chromosome addition lines under well-watered conditions [43] (Table 5), their amounts were lower under drought stress conditions in almost all of the lines (Table 3, S2 Fig). A similar trend was observed for the ratio of TOT-pentosan to β-glucan during drought, suggesting that the higher β-glucan content might be balanced by the lower TOT-pentosan content in wheat under drought stress. Wheat originally contains ten times more TOT-pentosan than β-glucan compared to *Aegilops*, which has almost equal quantities of these components. However, the increase in the β-glucan content was moderate in wheat and lower than expected after the addition of individual *Aegilops* chromosomes (S2 Fig). so the presence of *Aegilops* chromosome segments and/or genes might be necessary for a more significant increase in the β-glucan content of wheat. It should also be mentioned that the organs and milling fractions of wheat differ widely in their dietary fiber content and composition, but this cannot be discussed here as only the composition of the wholegrain was studied.

The pattern of TOTAL AXOS differed from that of TOT-pentosan under well-watered conditions [43] (Table 5), suggesting that pentosans other than AX may have influenced the results, though AX is the main component of TOT-pentosan. The discrepancy could also be due to differences in the digestibility of AX molecules in the two extraction protocols, as wholemeal samples contain various tissues. The TOTAL AXOS content of the *Aegilops* parents was lower than that of wheat under both drought and well-watered conditions, but there was no significant difference in the TOTAL AXOS content of the wheat addition lines except for line 7M^b^ under well-watered conditions.

The comparison of AX structure using enzymatic digestion showed that the proportion of US AXOS released was higher in *Aegilops* than in wheat under both well-watered and drought conditons (Table 3, Table 5, S4 Fig). This ratio was increased by the presence of chromosome 7M^b^ under well-watered conditions and by 3M^b^ during drought. The ratio of mono-substituted to di-substituted AXOS was also higher in the *Aegilops* species under both conditions, with chromosomes 2M^b^, 3M^b^ and 7M^b^ resulting in higher ratios under well-watered conditions. Under stress, both the US AXOS ratio and the water solubility of the pentosans decreased in most of the addition lines compared to wheat (though WE/WU increased), possibly due to the presence of longer chains and the higher number of crosslinks in the molecules.

It can therefore be concluded that the *Aegilops* parents, which had small kernels, had significantly higher contents of protein, β-glucan, TOTAL GOS, WE/WU-pentosan and US/M+D than wheat and that these properties were also manifested under drought stress conditions (Tables 3 and 5). Drought stress resulted in a general decrease in the TOT- and WE-pentosan content and TOT-pentosan/β-glucan ratio of the individual lines compared to wheat (Table 3), while these lines did not have lower pentosan content under well-watered conditions (Table 5).

In both treatments negative effects were observed for 1U^b^ and 1/6U^b^ on Glu/Gli, for 2U^g^ and 6M^g^ on WE-pentosan content, and for 7M^b^ on TOT-pentosan/β-glucan, and positive effects for 1M^g^ on UPP%, for 5U^g^, 7U^g^ and 7M^b^ on the β-glucan content, for 7M^g^ on TOTAL-GOS, and for 2M^b^, 3M^b^ and 7M^b^ on M/D.

Furthermore, drought stress resulted in higher protein content and TOT-pentosan/β-glucan ratio, and lower β-glucan content in many addition lines. Drought reduced both the β-glucan content and TOTAL-GOS of the 3M^g^ and 3M^b^ addition lines, while both TOT-pentosan and WE-pentosan increased in addition lines 1U^g^ and 2U^g^ due to drought.

In general, the grain composition of the *Aegilops* accessions were less affected by drought stress than that of the wheat parents (Table 4). While the protein content, TOT-pentosan, WE-pentosan, and TOT-pentosan/β-glucan content increased in both wheat genotypes, the β-glucan and TOTAL GOS content decreased. At the same time drought caused no change in the properties of *Ae*. *biuncialis*, while the Glu/Gli ratio and the WE-pentosan content increased and the β-glucan content decreased in *Ae*. *geniculata*.

The addition of *Aegilops* chromosomes could thus be expected to stabilize the compositional properties of the seed by mitigating the effect of drought stress.

The addition of chromosomes 4-5U^g^, 2M^g^, 5M^g^ and 7M^g^ resulted in greater stability in the protein content and composition under drought stress (Table 4), while chromosomes 3U^g^, 6U^g^, 1-2M^g^, 5M^g^, 1U^b^ and 3M^b^ stabilized he contents of both total-, WE-pentosans and β-glucan. Consequently, chromosomes 2M^g^ and 5M^g^ helped to stabilize both the protein and pentosan contents under drought stress. Since all the added chromosomes except 3U^g^ and 4U^g^ resulted in greater kernel size stability, it can be concluded that chromosomes 2M^g^ and 5M^g^ contributed to the stabilization of both kernel size and composition.

## Conclusions

Analysis on a series of wheat/*Aegilops* addition lines showed that compared to the wheat parents the *Aegilops* parents had higher protein and β-glucan contents, higher proportions of unsubstituted or less substituted AXOS, and more stable grain composition under drought stress conditions. The 2M^g^ and 5M^g^ chromosomes resulted in better grain composition stability under drought conditions, suggesting that *Aegilops biuncialis* and *Aegilops geniculata* could be used as genetic sources to improve the stability of grain composition and quality in wheat.

## Supporting information

S1 Fig Compositional properties of mature grains of two lines of bread wheat (cv. Chinese Spring and Mv9kr1 line), two *Aegilops* species (*Ae*. *geniculata*, *Ae*. *biuncialis*) and wheat-*Aegilops* chromosome addition lines under drought stress. a. thousand-kernel weight (TKW), b. protein content, c. glutenin to gliadin ratio (Glu/Gli), d. unextractable polymeric protein (UPP%).
* indicates values significantly higher than that of wheat (*T*. *aestivum*) based on LSD.
The control values were published by Rakszegi et al. (2017).
(LSD- least significant difference).
(JPG)

S2 Fig Compositional properties of mature grains of two lines of bread wheat (cv. Chinese Spring and Mv9kr1 line), two *Aegilops* species (*Ae*. *geniculata*, *Ae*. *biuncialis*) and wheat-*Aegilops* chromosome addition lines under drought stress. a. β-glucan, b. total (TOT) pentosan and c. water extractable (WE) pentosan content.
* indicates values significantly higher than that of wheat (*T*. *aestivum*) based on LSD.
The control values were published by Rakszegi et al. (2017).
(LSD- least significant difference).
(JPG)

S3 Fig Quantitative ratio of TOT-pentosan to β-glucan (a) and WE to WU-pentosan (b) in mature grains of two lines of bread wheat (cv. Chinese Spring and Mv9kr1 line), two *Aegilops* species (*Ae. geniculata*, *Ae. biuncialis*) and wheat-*Aegilops* chromosome addition lines under drought stress.
* indicates values significantly higher than that of wheat (T. aestivum) based on LSD.
The control values were published by Rakszegi et al. (2017).
(LSD- least significant difference, TOT- total, WE- water-extractable, WU- water-unextractable).
(JPG)

S4 Fig Quantity of arabinoxylan units in mature grains of two lines of bread wheat (cv. Chinese Spring and Mv9kr1 line), two *Aegilops* species (*Ae*. *geniculata*, *Ae*. *biuncialis*) and wheat-*Aegilops* chromosome addition lines under drought stress after enzymatic fingerprinting. a. quantity of β-glucan units, b. ratio of DP3 to DP4 units, c. TOT-AXOS, d. ratio of Unsubstituted AXOS (US) to monosubstituted (M) + disubstituted (D) AXOS, e. M/D ratio.
* indicates values significantly higher than that of wheat (*T*. *aestivum*) based on LSD.
The amounts of monosubstituted (M), disubstituted (D), unsubstituted (US) and total (TOT) AXOS were calculated as decribed in Rakszegi et al. (2017).
(AXOS- arabinoxylan oligosaccharide, DP- degree of polymerization, GOS- glucooligosaccharides, LSD- least significant difference, TOT- total).
(JPG)

## Abbreviations

A/X: ratio of arabinose to xylose
AX: arabinoxylan
AXOS: arabinoxylan oligosaccharides
DP: degree of polymerization
D: disubstituted AXOS
Gli: gliadin
Glu: glutenin
GMP: glutenin macropolymers
GOS: glucooligosaccharides
HMW: high molecular weight
HP-AEC: high performance anion exchange chromatography
LSD: least significant difference
M: monosubstituted AXOS
SE-HPLC: size exclusion high performance liquid chromatography
SWC: soil water content
TKW: thousand-kernel weight
TOT-AX: total arabinoxylan
TOT: total AXOS
UPP: unextractable polymeric protein
US: unsubstituted AXOS
WE: water-extractable
WU: water-unextractable
