# Supplementary figures and images for: Drought stress affects the protein and dietary fiber content of wholemeal wheat flour in wheat/Aegilops addition lines
Source: PLoS One. 2019 Feb 5;14(2):e0211892. doi: 10.1371/journal.pone.0211892 (PMC6363227; doi:10.1371/journal.pone.0211892)

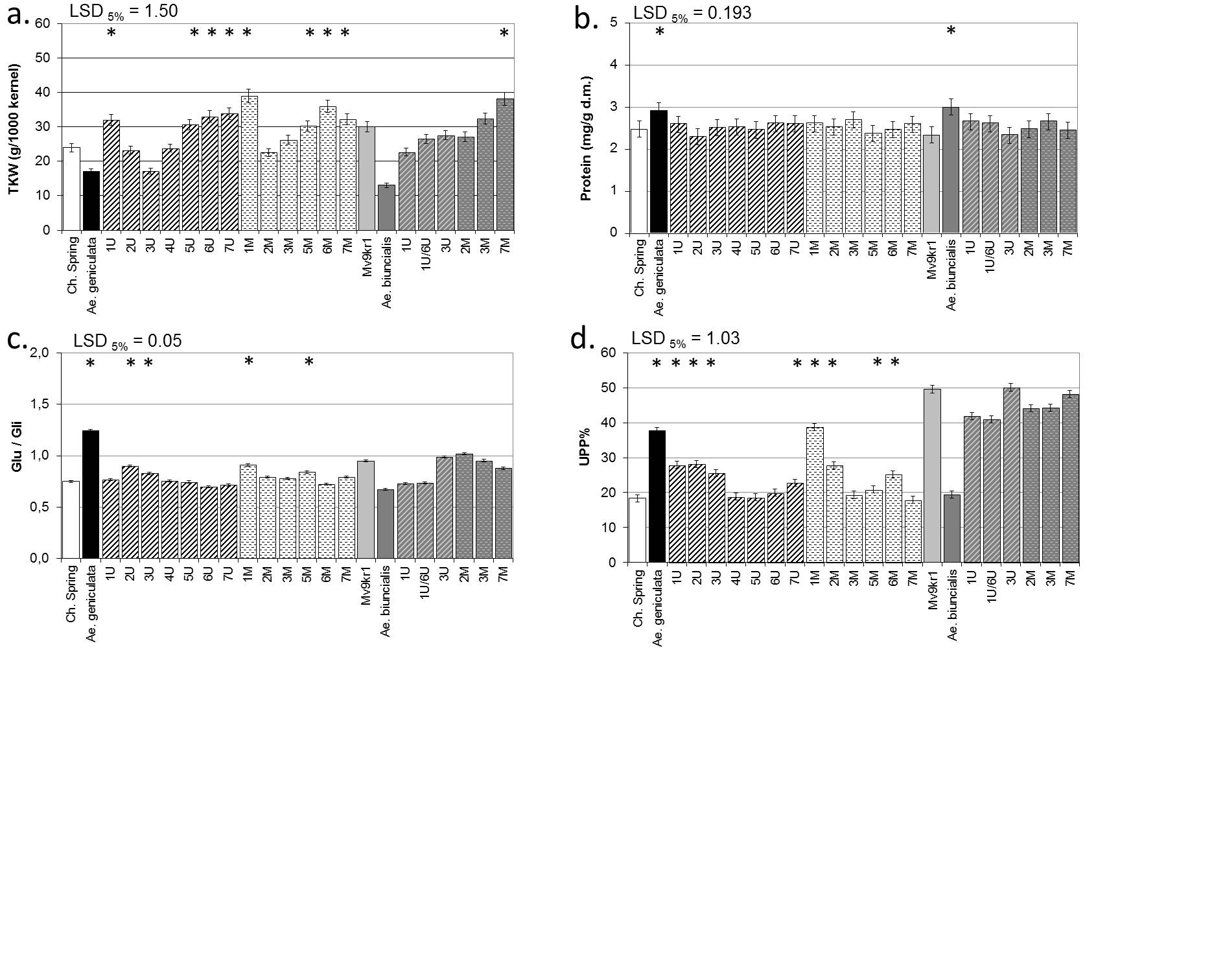

Supplement: S1 Fig — a. thousand-kernel weight (TKW), b. protein content, c. glutenin to gliadin ratio (Glu/Gli), d. unextractable polymeric protein (UPP%). * indicates values significantly higher than that of wheat (T. aestivum) based on LSD. The control values were published by Rakszegi et al. (2017). (LSD- least significant difference). (JPG) [file pone.0211892.s001.jpg]

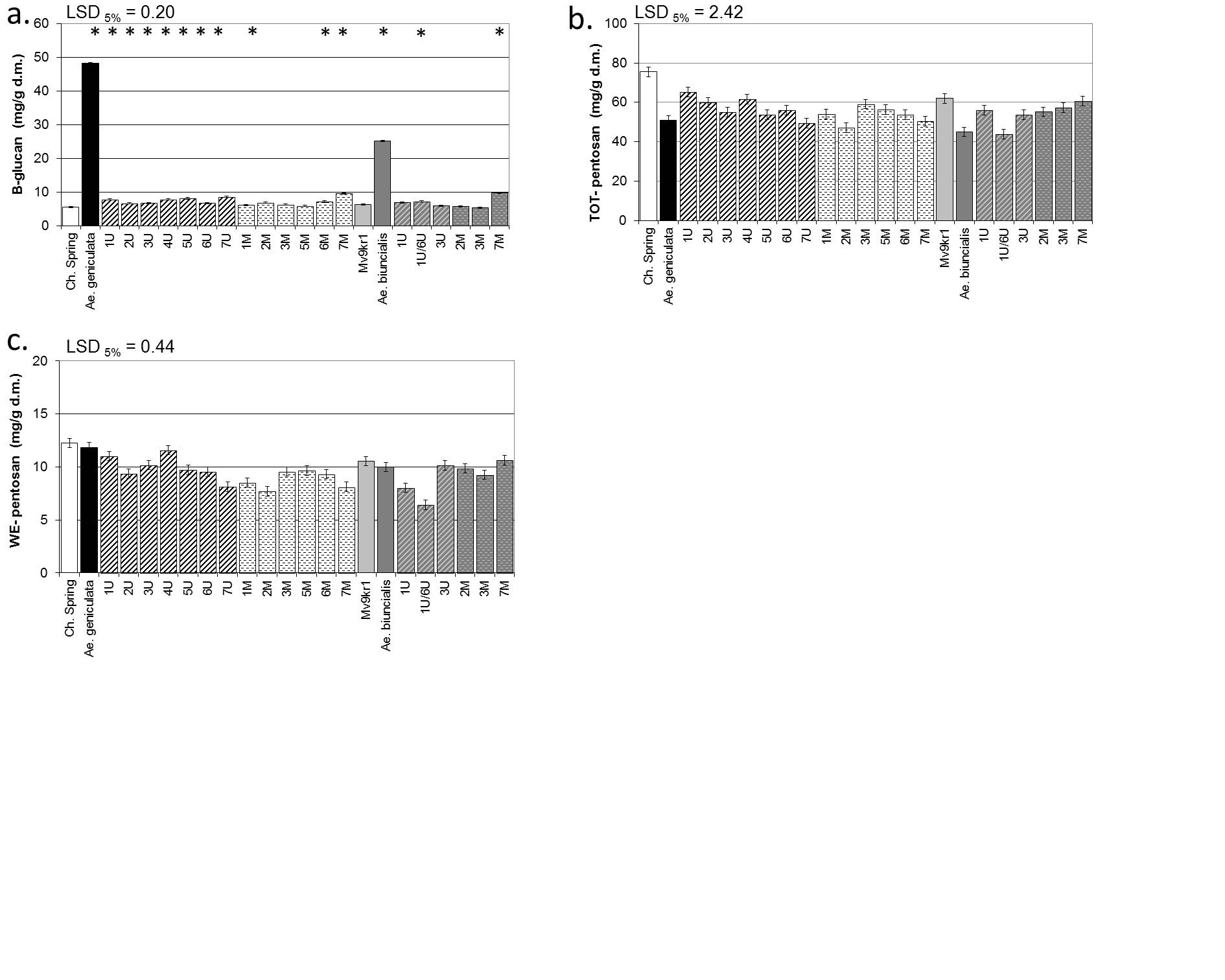

Supplement: S2 Fig — a. β-glucan, b. total (TOT) pentosan and c. water extractable (WE) pentosan content. * indicates values significantly higher than that of wheat (T. aestivum) based on LSD. The control values were published by Rakszegi et al. (2017). (LSD- least significant difference). (JPG) [file pone.0211892.s002.jpg]

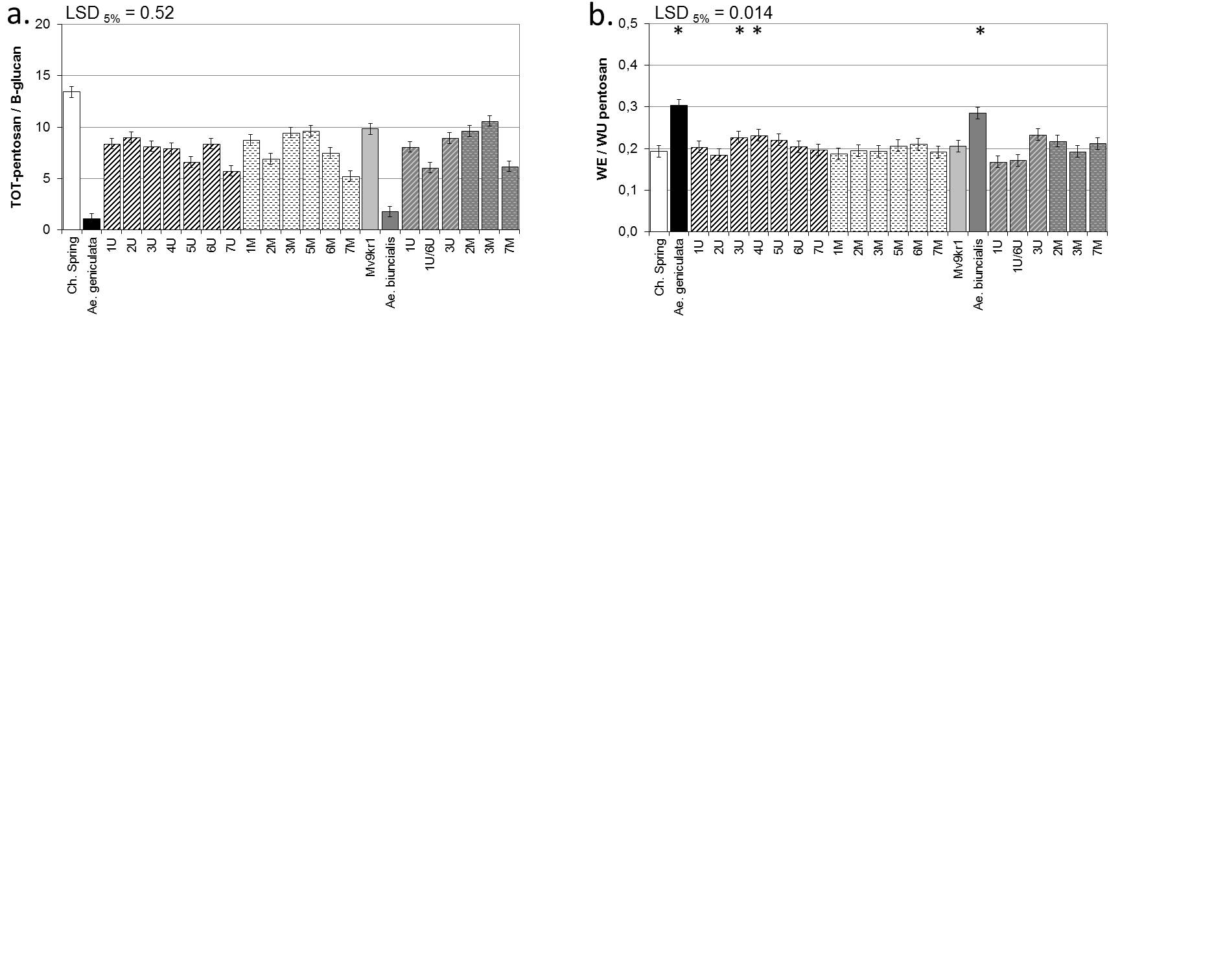

Supplement: S3 Fig — Quantitative ratio of TOT-pentosan to β-glucan (a) and WE to WU-pentosan (b) in mature grains of two lines of bread wheat (cv. Chinese Spring and Mv9kr1 line), two Aegilops species (Ae. geniculata, Ae. biuncialis) and wheat-Aegilops chromosome addition lines under drought stress. * indicates values significantly higher than that of wheat (T. aestivum) based on LSD. The control values were published by Rakszegi et al. (2017). (LSD- least significant difference, TOT- total, WE- water-extractable, WU- water-unextractable). (JPG) [file pone.0211892.s003.jpg]

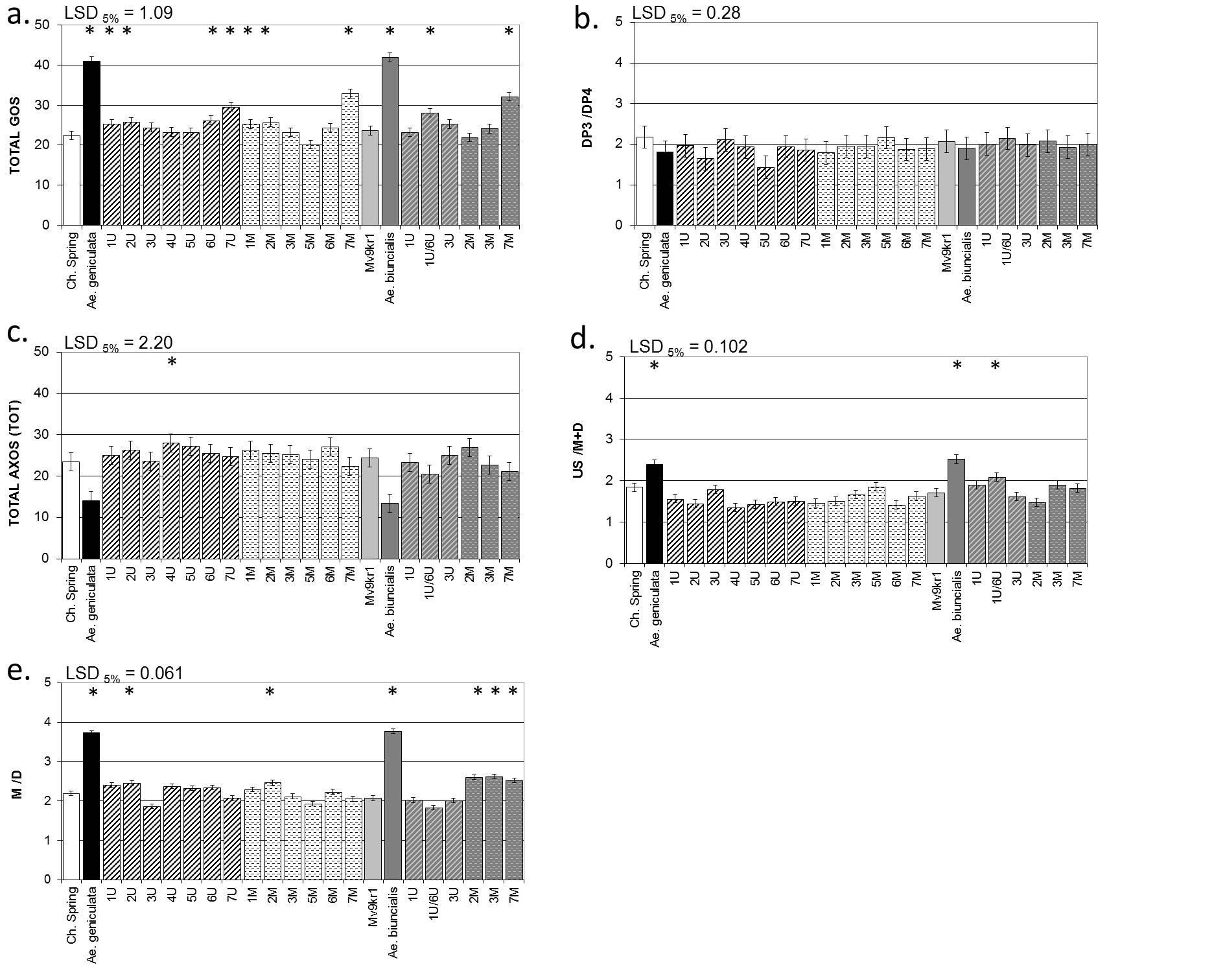

Supplement: S4 Fig — a. quantity of β-glucan units, b. ratio of DP3 to DP4 units, c. TOT-AXOS, d. ratio of Unsubstituted AXOS (US) to monosubstituted (M) + disubstituted (D) AXOS, e. M/D ratio. * indicates values significantly higher than that of wheat (T. aestivum) based on LSD. The amounts of monosubstituted (M), disubstituted (D), unsubstituted (US) and total (TOT) AXOS were calculated as decribed in Rakszegi et al. (2017). (AXOS- arabinoxylan oligosaccharide, DP- degree of polymerization, GOS- glucooligosaccharides, LSD- least significant difference, TOT- total). (JPG) [file pone.0211892.s004.jpg]
